# Gasdermin D-mediated pyroptosis: mechanisms, diseases, and inhibitors

**DOI:** 10.3389/fimmu.2023.1178662

**Published:** 2023-05-18

**Authors:** Zhen Dai, Wan-Cong Liu, Xiao-Yi Chen, Xiao Wang, Jun-Long Li, Xiang Zhang

**Affiliations:** ^1^ Sichuan Industrial Institute of Antibiotics, School of Pharmacy, Chengdu University, Chengdu, China; ^2^ Key Laboratory of Drug Quality Control and Pharmacovigilance, Ministry of Education, China Pharmaceutical University, Nanjing, China

**Keywords:** GSDMD, pyroptosis, inflammasome, GSDMD pores, mechanism, GSDMD inhibitors

## Abstract

Gasdermin D (GSDMD)-mediated pyroptosis and downstream inflammation are important self-protection mechanisms against stimuli and infections. Hosts can defend against intracellular bacterial infections by inducing cell pyroptosis, which triggers the clearance of pathogens. However, pyroptosis is a double-edged sword. Numerous studies have revealed the relationship between abnormal GSDMD activation and various inflammatory diseases, including sepsis, coronavirus disease 2019 (COVID-19), neurodegenerative diseases, nonalcoholic steatohepatitis (NASH), inflammatory bowel disease (IBD), and malignant tumors. GSDMD, a key pyroptosis-executing protein, is linked to inflammatory signal transduction, activation of various inflammasomes, and the release of downstream inflammatory cytokines. Thus, inhibiting GSDMD activation is considered an effective strategy for treating related inflammatory diseases. The study of the mechanism of GSDMD activation, the formation of GSDMD membrane pores, and the regulatory strategy of GSDMD-mediated pyroptosis is currently a hot topic. Moreover, studies of the structure of caspase-GSDMD complexes and more in-depth molecular mechanisms provide multiple strategies for the development of GSDMD inhibitors. This review will mainly discuss the structures of GSDMD and GSDMD pores, activation pathways, GSDMD-mediated diseases, and the development of GSDMD inhibitors.

## 1 Introduction

The term “pyroptosis” was first proposed in 2001 and consists of “pyro” and “ptosis”, which represent the features of inflammation (fire or fever) and programmed cell death (falling), respectively (1). Due to the discovery of diverse pyroptosis-executing proteins, pyroptosis has been redefined as a form of programmed necrosis mediated by gasdermin proteins with the characteristics of cell swelling, membrane rupture, and the release of cellular contents (2, 3). Gasdermins belong to the pore-forming protein family and consist of six gasdermin proteins, including gasdermins A-E and DFNB59 (4). Among them, DFNB59 is not associated with the formation of membrane pores and pyroptosis (5). With the exception of DFNB59, the members of the gasdermin family have two domains: an N-terminal domain and a C-terminal domain linked by a flexible peptide. Upon activation, the cleaved N-terminal domain is responsible for inducing pyroptosis (6–8). This review focuses on GSDMD, the most extensively studied executive pyroptosis-executing protein with the clearest mechanism. More detailed discussions of other members of the gasdermin family can be found in other reviews (3, 4, 9, 10).

GSDMD serves as a direct substrate of inflammatory caspases, including caspase-1/4/5 and murine caspase-11, and is cleaved into an active N-terminal domain (GSDMD-NT) upon canonical or noncanonical inflammasome activation induced by exogenous stimuli or endogenous injuries (6). Recent studies have demonstrated that caspase-8, which is responsible for apoptosis, is also involved in GSDMD activation (11, 12). GSDMD-NT oligomerizes and forms membrane pores, leading to the release of inflammatory factors such as IL-1β and IL-18, as well as non-selective ion fluxes (13–16). Massive membrane pore formation compromises membrane integrity, causing lytic cell death and the release of cytoplasmic contents that amplify inflammatory signals.

While GSDMD-induced pyroptosis has been shown to protect the host against bacterial infection (17, 18), numerous studies have demonstrated that abnormal GSDMD activation causes severe inflammatory cascades such as disruption of ionic homeostasis, organelle dysfunction, cell lysis, and sustained release of inflammatory cytokines. Abnormal GSDMD activation can cause persistent inflammation, which has been implicated in various inflammatory diseases, including ischemic stroke (19, 20), familial Mediterranean fever (FMF) (21), neonatal-onset multisystem inflammatory disease (NOMID) (22), experimental autoimmune encephalitis (EAE) (23), sepsis (24, 25), nonalcoholic fatty liver disease (NAFLD) (26), cancer (27–29), human immunodeficiency virus (HIV) infection (30), neurodegenerative diseases such as Alzheimer’s disease (AD) (31, 32) and Parkinson’s disease (PD) (33). Moreover, cell pyroptosis has been shown to play an important role in a series of clinical symptoms caused by severe acute respiratory syndrome-related coronavirus 2 (SARS-CoV-2) infection. Excessive inflammation and cytokine storm are the main causes of tissue damage and organ failure in COVID-19. GSDMD serves as a regulatory protein upstream of cytokine storm and is thus a promising target for the treatment of COVID-19 (34). Given the important role of GSDMD in pyroptosis and inflammatory disorders, we summarize the structure and activation mechanisms of GSDMD and focus on the discovery of GSDMD inhibitors in this review.

## 2 GSDMD and membrane pores

### 2.1 Structure of GSDMD

**Figure 1:**
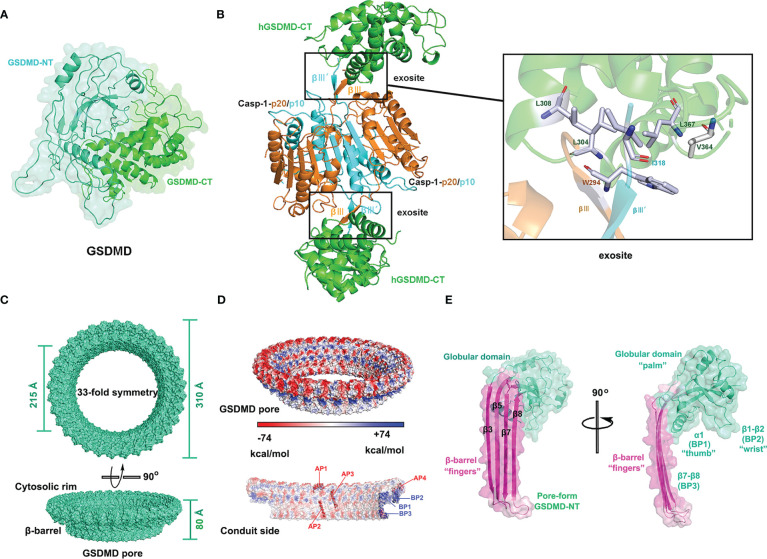
Structures of GSDMD, caspase-1-GSDMD complex, and GSDMD pore. **(A)** Crystal structure of human GSDMD (PDB: 6N9O). **(B)** Crystal structure of the caspase-1 (C285A) p20/p10-hGSDMD-CT complex (PDB: 6KN0) and the binding surface. **(C)** Cryo-EM structure of human GSDMD pore (PDB:6VFE). **(D)** Electrostatic surface (-74 to +74 kcal/mol) of the GSDMD pore. The acidic patches (APs) and basic patches (BPs) of the GSDMD pore are marked in red and blue, respectively. **(E)** Cryo-EM structure of pore-formed GSDMD-NT, resembling a human left hand, with β-barrel (β3, β5, β7, and β8) as “fingers”, the α1 helix as “thumb”, the globular domain as “palm”, and the β1-β2 sheets as “wrist”.

The full-length GSDMD protein has two characteristic domains: a pore-forming GSDMD-NT (also known as p30) and an inhibitory C-terminal domain (GSDMD-CT, also called p20), as shown in **Figure 1A**. These two domains are linked by a flexible loop that contains the GSDMD activation site, ^272^FLT**D** ^275^ of human GSDMD (hGSDMD) or ^273^LLS**D** ^276^ of murine GSDMD (mGSDMD) (7, 35). In resting cells, GSDMD-NT binds to GSDMD-CT through intramolecular interactions, leading to full-length GSDMD in autoinhibition. The cleavage of GSDMD is often, but not always, performed by inflammation-activated caspase-1 or LPS-stimulated caspase-4/5/11, leading to the release of GSDMD-NT and the subsequent formation of GSDMD membrane pores (6). In addition to activating GSDMD, caspase-1, a typical member of the inflammatory caspase family, also cleaves pro-IL-1β and pro-IL-18 into their active forms to promote their maturation and secretion (36, 37). A recent report has shown that caspase-4/5 cleave IL-18 at Asp^36^ to generate the active species, while caspase-11 is unable to process IL-18 (38). However, caspase-4/5/11 cannot activate but cleave IL-1β at Asp^27^ to form an inactive p27 fragment (38). GSDMD is cleaved by caspase-1/4/5/11 at the tetrapeptide region ^272^FLT**D** ^275^|G^276^ (^273^LLS**D** ^276^|G^277^) for mouse GSDMD), inducing subsequent pyroptosis and inflammatory responses (6, 39).

In 2020, Wang and colleagues analyzed the high-resolution crystal structures of CASP-GSDMD complexes, revealing the interaction between the exosites of caspases with GSDMD-CT (25). Upon inflammasome activation or LPS stimulation, pro-caspases self-cleave into activated caspase heterodimers, which further dimerize to form tetramers. Two symmetrical protruding intermolecular β-sheets, βIII and βIII’, are formed on the tetrameric interface independent of the cysteine active sites of inflammatory caspases. The βIII and βIII’, stretching outside of the tetramer, insert like a key into a hydrophobic pocket of GSDMD-CT to form 2:2 enzyme-substrate complexes with GSDMD-CT. For the complex of caspase-1-GSDMD-CT (PDB: 6KN0), two key amino acid residues of caspase β-sheets, Trp^294^ on βIII and Ile^318^ on βIII’ of caspase-1, form hydrophobic interactions with the GSDMD-CT groove (Leu^304^, Leu^308^, Val^364^, and Leu^367^), as shown in **Figure 1B** (25). Mutations in either the key amino acids of the βIII/βIII’ strands or the hydrophobic residues of GSDMD-CT disrupted the binding of caspases to GSDMD-CT, thereby inhibiting GSDMD cleavage and pyroptosis. The crystal structure of the caspase-1-mGSDMD complex (PDB: 6VIE) has been analyzed by Xiao’s group, which further revealed the structural basis of the interaction between caspase-1 and full-length GSDMD (40). Activated human caspase-1 forms two distinct but adjacent binding interfaces with the mGSDMD linker region and mGSDMD-CT domain, respectively, but does not interact with mGSDMD-NT. On the one hand, caspase-1 binds to the mGSDMD cleavage site at the disordered linker region. On the other hand, activated caspase-1 forms a protruding double β-strand that interacts with the GSDMD-CT domain via hydrophobic interactions and hydrogen bonding, as reported by Wang (25, 40). Mutations in the exosite-binding residues of mGSDMD-CT (L306A, L310A, L361A, V367A, and L370A), as well as in caspase-1 (W294A, I318E, and K320E), have been discovered to prevent the formation of the caspase-1-mGSDMD complex and subsequent GSDMD cleavage. The dual-interface engagement of caspase-1 with GSDMD suggests that the GSDMD-CT domain not only has an inhibitory function but also recruits caspase proteins, providing a platform for GSDMD activation.

### 2.2 Structure of GSDMD pores

Upon cleaving of the linked region by inflammatory caspases, the GSDMD-CT dissociates, exposing the β1-β2 loop which then binds to acidic lipids. The hydrophobic tip, hGSDMD ^48^WFW^50^ or mGSDMD ^50^FW^51^, of the β1-β2 loop anchors into the lipid bilayer, and the surrounding basic residues interact with acidic lipids (41). Mutational studies have shown that hydrophobic amino acid mutations in the β1-β2 loop of GSDMD, including W48E and W50E of hGSDMD and a double mutant F50G/W51G of mGSDMD, impair pore formation (35, 41). In addition, basic amino acid mutations in the β1-β2 loop of GSDMD, such as basic patch 2 (BP2) (R42E/K43E/K51E/R53E) and BP3 (K204E/R174E), also inhibit or even eliminate the formation of GSDMD membrane pores (41).

Hao Wu and her collaborators analyzed the cryo-EM structures of GSDMD pores and GSDMD prepores (41). GSDMD pore is 10-20% larger than the GSDMA3 pore and comprises 31-34 subunits (41, 42). GSDMD pores with 33 subunits have the highest resolution (**Figure 1C**). The GSDMD pore consists of a globular region and a transmembrane region, the latter of which is also called as β-barrel and is composed of 132 β-strands (41). As shown in **Figure 1E**, each pore-forming GSDMD-NT subunit has a structure resembling a human left hand, with “fingers” (β-barrel) inserted into plasma membranes, one “thumb” (α1 helical, also known as basic patch 1(BP1)), a “palm” (globular domain), and a “wrist” (β1-β2 loop, BP2). However, the conformation of GSDMD prepores differs considerably from that of GSDMD pores. The subunit of GSDMD prepores lacks a β-barrel, similar to the inhibited globular N-terminal domain in full-length GSDMD. The structural analysis of GSDMD prepores indicated that GSDMD-NT first oligomerized and subsequently underwent conformational changes after being cleaved by inflammatory caspases. The pore-forming precursor may be an intermediate in the process of GSDMD pore assembly, but the precise method by which GSDMD pores are assembled in cells is still unknown (41).

In hyperactivated cells without pyroptosis, the inner diameter of GSDMD transmembrane pores determines the release of intracellular proteins, allowing the inflammatory cytokines IL-1β and IL-18 to be released while larger proteins such as high-mobility group box 1 (HMGB1, 150 kDa) and LDH (140 kDa) are filtered out (13). Thus, the release of LDH is considered a symbolic event for cell pyroptosis. Although the mature and precursor forms of IL-1 proteins with similar size (~ 4 nm) are significantly smaller than GSDMD membrane pores (~20 nm), the transport rates of the two forms differ. GSDMD membrane pores preferentially release mature IL-1 over pro-IL-1, suggesting the involvement of other factors in the transport process. The cryo-electron microscopy study showed different charge distributions in the structure of GSDMD transmembrane pores. As **Figure 1D** shows, the GSDMD pore on the membrane-facing side (parallel to the cell membrane) has three positively charged patches or basic patches (BPs) that interact with acidic lipids, whereas the conduit (β-barrel) inserted into the cell membrane with acid patches (APs) is predominantly negatively charged. GSDMD pores filter against negatively charged pro-IL-1β via electrostatic interaction, while mature IL-1β without an acidic domain is released into the extracellular environment (41). Recently, Wang’s group demonstrated that the selectivity of GSDMD pores for IL-1β can be regulated by modifying the degree of lipid binding of GSDMD and salt concentration (43). Enhanced interaction between GSDMD and lipids increases the selectivity of the membrane pores for the release of mature IL-1β, whereas a high concentration of salt decreases the selectivity, allowing more non-selective release of pro-IL-1β (43).

## 3 GSDMD activation pathways

### 3.1 Inflammasomes-mediated GSDMD activation pathway

**Figure 2:**
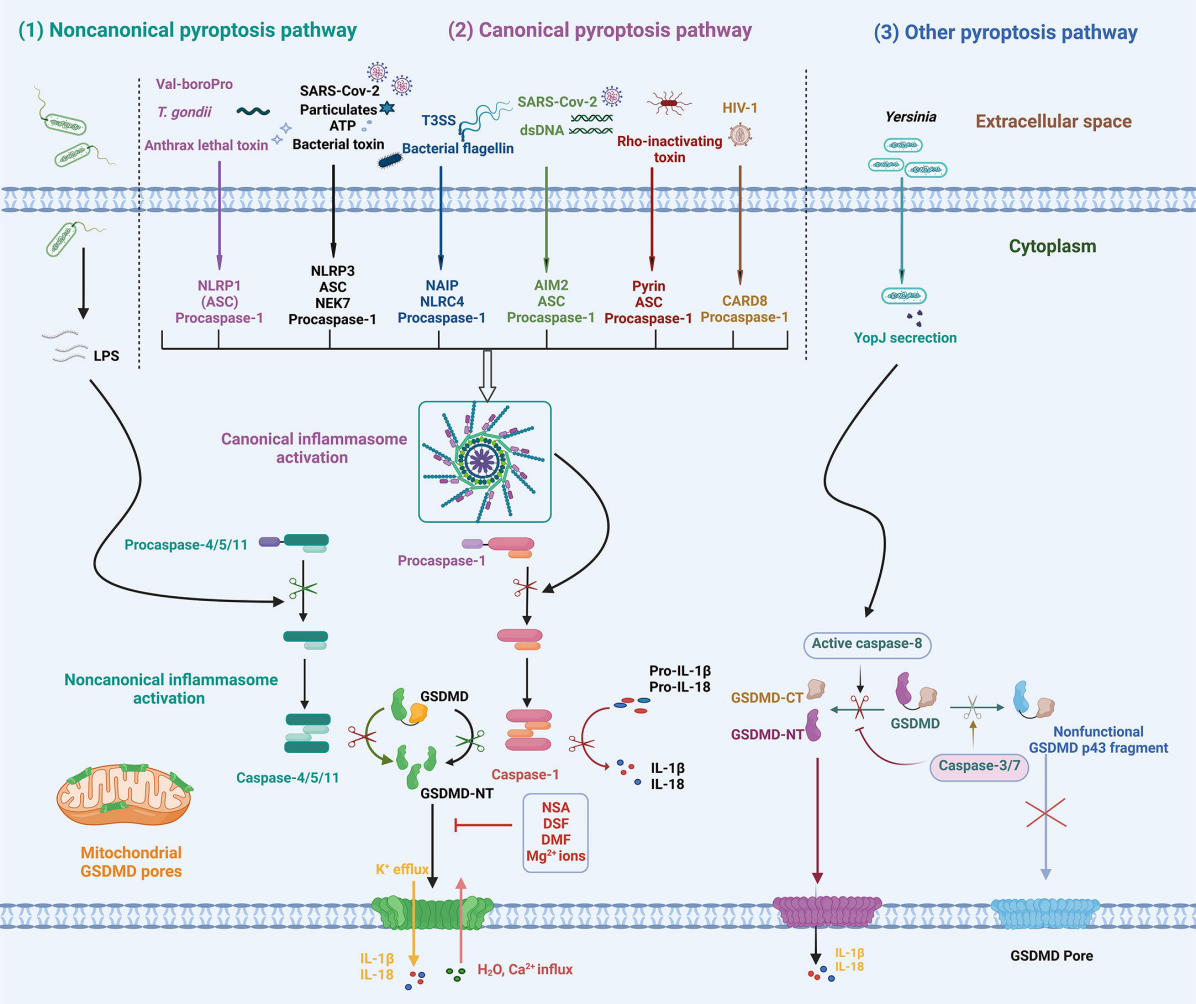
Schematic diagram of the mechanisms of GSDMD activation and pyroptosis. In response to various PAMPs or DAMPs, inflammatory caspase-1/4/5/11 is activated, leading to GSDMD cleavage and activation. In addition, apoptotic caspase-8 activation induced by *Yersinia* infection cleaves GSDMD into GSDMD-NT. The activated GSDMD-NT oligomerizes and inserts into membranes, forming GSDMD pores that lead to ion fluxes, inflammatory cytokine release, and pyroptosis. NLRP1, NOD-, LRR- and pyrin domain-containing protein 1; NLRP3, NOD-, LRR- and pyrin domain-containing protein 3; NLRC4, NOD-like receptor family CARD-containing 4; AIM2, absent in melanoma 2; CARD-8, caspase activation and recruitment domain 8; ASC, apoptosis-associated speck-like protein containing a CARD; LPS, lipopolysaccharide; *T*. *gondii*, *Toxoplasma gondii*; T3SS, type III secretion system; dsDNA, double-stranded DNA; YopJ, *Yersinia* effector protein, also called YopP in *Yersinia enterocolitica*; NSA, necrosulfonamide; DSF, disulfiram; DMF, dimethyl fumarate.

Since the discovery of the pyroptotic function of GSDMD proteins, various regulatory pathways of GSDMD-mediated pyroptosis have been revealed. These pathways can be classified into canonical, noncanonical, and other pyroptosis pathways (2, 6, 7, 39, 44), as shown in **Figure 2**. Inflammasome-mediated GSDMD cleavage and subsequent cell lysis are the major regulatory pathways of pyroptosis. In the innate immune system, germline-encoded pattern recognition receptors (PRRs) recognize and detect pathogen-associated molecular patterns (PAMPs) and danger-associated molecular patterns (DAMPs). Cytoplasmic receptor proteins of PRR, mainly including the nucleotide-binding oligomerization domain (NOD)-like receptors (NLRs) family, absent in melanoma 2 (AIM2), pyrin protein, and CARD8, are activated by intracellular PAMPs or DAMPs and assemble to form canonical inflammasomes. A typical inflammasome is a multimeric complex generally composed of a sensor protein, an adaptor protein (ASC, apoptosis-associated speck-like protein containing a CARD), and an effector protein (caspase-1). The recognition and detection of PAMP or DAMP by PRRs initiate the inflammasome signaling pathway. Activated receptor proteins oligomerize and bind to ASC, and then recruit the effector protein pro-caspase-1 to form canonical inflammasome complexes. Pro-caspase-1 autoproteolyzes into active caspase-1, inducing a cascade of inflammatory responses. ASC oligomerizes to form insoluble polymers termed ASC speck during inflammasome activation, which serves as a signaling platform for significantly amplifying caspase-1 activation (45, 46). However, ASC is dispensable for the formation of some inflammasomes. PRRs containing a CARD domain, such as NLRP1b and NLRC4, can directly bind to pro-caspase-1, but the activity of caspase-1 is significantly enhanced in the presence of ASC (47). Activation of caspase-1 in the absence of ASC cleaves GSDMD and induces pyroptosis, but it cannot effectively cleave inflammatory cytokines such as IL-18 and IL-1β.

In the noncanonical pathway, inflammatory caspases (caspase-4/5/11) serve as receptor proteins and directly respond to intracellular LPS without requiring the recruitment of receptor proteins or the adaptor protein ASC. The CARD domain of pro-caspase-11 in rodents (pro-caspase-4/5 in humans) directly binds to the lipid A of LPS, resulting in conformational change, oligomerization, and autoproteolysis of pro-caspase-4/5/11 to form noncanonical inflammasomes (48–50). The activation of noncanonical inflammasomes induces GSDMD cleavage and pyroptosis, which play an important role in the pathogenesis of bacterial infections and sepsis (14, 51, 52). Caspase-4/5/11 are unable to cleave pro-IL-1β into active forms, whereas caspase-4/5, instead of caspase-11, has been found to cleave pro-IL-18 into mature IL-18 (38, 53). Further studies are necessary to reveal the molecular basis of IL-1β and IL-18 recognition and cleavage by inflammatory caspases in noncanonical pathways. Moreover, caspase-4/5/11 can also activate NLRP3 inflammasome through downstream events such as GSDMD-NT pore-related K^+^ efflux, leading to the maturation and secretion of IL-1β and IL-18 (54–57).

Considering multiple regulatory pathways, GSDMD activation and related downstream effects are generally divided into the following key steps (1): activation of effector caspases; (2) GSDMD cleavage and activation; (3) GSDMD oligomerization and pore formation; and (4) downstream inflammation (hyperactivation or pyroptosis). The crucial event in GSDMD activation is the cleavage of GSDMD at the active site ^272^FLT**D** ^275^ (^273^LLS**D** ^276^ for mGSDMD) by inflammatory caspase-1/4/5/11, resulting in GSDMD-NT and GSDMD-CT fragments (2, 3, 6, 39, 58). The active GSDMD-NT fragments oligomerize and bind to acidic lipids in the plasma membranes, forming transmembrane pores that allow ion flux, water influx, and selective cytokine release. Further cell membrane rupture releases massive cellular contents, including IL-1β, IL-18, HMGB1, ATP, and LDH, causing severe inflammatory responses. In addition, GSDMD-NT also binds to mitochondrial cardiolipin to form mitochondrial GSDMD pores that release mitochondrial ROS (mtROS) and mitochondrial DNA (mtDNA), leading to mitochondrial dysfunction (2, 59, 60).

### 3.2 Other GSDMD regulatory mechanism

In addition to inflammatory caspases, several studies have revealed apoptotic caspases also play a role in GSDMD regulation. During apoptosis, caspase-3/7 specifically blocks pyroptosis by cleaving GSDMD at hGSDMD Asp^87^ (mGSDMD Asp^88^) independent of the cleavage site and the exosite-binding pocket to form an inactive p43 fragment, which is a general feature of apoptosis in cells expressing GSDMD (61). Caspase-3/7 activation occurs after caspase-1-driven GSDMD cleavage and pyroptosis, serving as a negative feedback mechanism to inhibit excessive pyroptosis and inflammation (44, 61). These findings suggest a complex interplay between cell death pathways in the innate immune system. Pyroptotic stimuli can activate apoptosis signaling through multiple mechanisms, while apoptotic stimuli specifically prevent or even deactivate pyroptosis.

The activation pathway of caspase-8, responsible for apoptosis, was successively described by Lien and Poltorak as a complementary mechanism for GSDMD activation and pyroptosis (11, 12). GSDMD and GSDME were significantly cleaved upon the activation of the receptor-interacting serine-threonine protein kinase 1 (RIPK1)-caspase-8 pathway. In addition to apoptosis, pyroptotic morphological features such as plasma membrane rupture were also observed in bone marrow-derived macrophages (BMDMs) infected by *Yersinia pseudotuberculosis* or treated with LPS and 5z-7-oxozaeenol (5z7), a transforming growth factor-β-activated kinase 1 (TAK1) inhibitor (11, 12). The deficiency of GSDMD delays membrane rupture and converts cell pyroptosis to apoptosis (12). This suggests that TAK1 inhibition-mediated activation of caspase-8 induces both apoptosis and pyroptosis, which breaks the cognition that only inflammatory caspases activate GSDMD. Broz’s group further demonstrated that TAK1 inhibition or inhibitors of apoptosis proteins (IAPs) depletion promotes activation of caspase-8, resulting in the cleavage of GSDMD at the same active site as caspase-1 and cell pyroptosis (62). In addition, caspase-8-mediated inflammatory regulatory functions can be blocked by caspase-3-dependent cleavage at mGSDMD Asp^88^, which is essential for caspase-8-dependent apoptosis (62). However, a separate study found that TLR priming in TAK1-deficient cells triggers caspase-8 activation and GSDMD-dependent pyroptosis independently of the kinase activity of RIPK1 (63). Therefore, the pathways by which the presence and absence of TAK1 regulate RIPK1 function and subsequent cell death need to be further explored.In a separate study, Poltorak et al. found that cellular FLICE-like inhibitory protein (cFLIP) downstream of TAK1 protein plays a role in inflammation and cell death by regulating the formation of apoptosis-related complex II (also called ripoptosome) consisting of RIPK1, TRADD, Fas-associated death domain (FADD), and caspase-8 (64). A low level or deficiency of cFLIP_L_ promotes the formation of complex II, auto-processing caspase-8 to form an active homodimer. The activated caspase-8 cleaves GSDMD to induce pyroptosis and activates the NLRP3 inflammasome, resulting in the maturation and release of IL-1β (64). In 2021, further studies revealed the molecular mechanism of the RIPK1-caspase-8-GSDMD pathway activated by TAK1 inhibition (65). Through a genome-wide CRISPR-Cas9 screen, Liu et al. identified the Rag-Ragulator complex, consisting of RagA, RagC, and Lamtor1-5, as a key factor in *Yersinia* infection and pyroptosis initiation. In response to pathogenic *Yersinia* or the TAK1 inhibitor 5z7, the FADD-RIPK1-pro-caspase-8 complex is recruited to the lysosomal membrane via the Rag-Ragulator complex (65). The binding of FADD-RIPK1-caspase-8 to the Rag-Ragulator complex licenses RIPK1 phosphorylation, caspase-8 activation, cleavage of GSDMD and GSDME, and subsequent pyroptosis, which depends on Rag GTPase activity and Rag-Ragulator lysosomal binding but not on rapamycin complex 1 (mTORC1) (65). It is worth mentioning that Kagan and his collaborators also successively confirmed the important role of the Rag-Ragulator complex in GSDMD membrane pores and cell pyroptosis in the same year (66). In contrast to Liu’s conclusion, Kagan et al. found that the activity of the downstream effector mTORC1 is required for the formation of GSDMD-NT pores. The findings showed that the Rag-Ragulator-mTORC1 played an important role in GSDMD pore formation and pyroptosis by promoting GSDMD-NT oligomerization without affecting the cleavage of GSDMD or the anchoring of GSDMD to the plasma membrane. Furthermore, the activation of the Rag-Ragulator complex leads to mitochondrial damage and ROS production in macrophages, which triggers GSDMD-NT oligomerization, membrane pore formation, and pyroptosis (66). Overall, caspase-8 is a molecular switch that controls multiple cell death processes, including apoptosis, necroptosis, and pyroptosis, and performs various functions in the cell death process. In the case of compromised apoptosis and necroptosis, caspase-8 activates the inflammasome and induces pyroptosis as an alternative death mechanism (67, 68).

Besides the regulation of GSDMD by caspases, other pathways for GSDMD activation have also been found (69–71). In neutrophils, ELANE, a specific neutrophil serine protease (NSP) in cytoplasmic granules, cleaves hGSDMD at Cys^268^ and mGSDMD at residue Val^251^, suggesting that GSDMD is recognized by ELANE probably through tertiary structures rather than specific amino acid sequences (69). The ELANE-mediated cleavage of GSDMD yields ELANE-derived NT fragments (GSDMD-eNT) with slightly smaller molecular weights but comparable activity to common GSDMD-NT, contributing to neutrophil death (69, 71). Cathepsin G is another NSP that activates GSDMD and induce pyroptosis (70). Cathepsin G cleaves Leu^274^ of mGSDMD to generate the specific nitrogen-terminal domain p30, which is blocked by Serpinb1 and Serpinb6, the key survival factors in neutrophils and monocytes, suggesting that tight regulation of cell death pathways and inflammatory responses (70, 72). In addition, Zika virus (ZIKV), an oncolytic virus targeting glioblastoma (GBM) cells, induces cytolysis by caspase-independent pyroptosis. ZIKV NS2B3 protease specifically cleaves GSDMD at residue Arg^249^ into an active N-terminal fragment for pyroptosis, thus inducing infected and nearby uninfected cell death (73, 74).

Posttranslational modifications of GSDMD, including succination, palmitoylation, ubiquitination, and oxidation, have been shown to play a critical role in the regulation of GSDMD activation. Fitzgerald et al. have revealed that dimethyl fumarate (DMF) or accumulation of endogenous fumarate modifies GSDMD irreversibly at key cysteine residues to generate 2-(succinyl)-cysteine (75). This GSDMD succination blocks the interaction of GSDMD with caspases, GSDMD processing, and oligomerization, thereby preventing GSDMD pore formation and pyroptosis (75). Recently, Luo et al. and Wu’s group independently discovered that palmitoylation of GSDMD, a reversible post-translational lipid modification, plays an important role in the biological processes of membrane translocation and pore formation (76, 77). Full-length GSDMD and GSDMD-NT are palmitoylated at Cys^191^/Cys^192^ (human/mouse), which leads to membrane translocation of GSDMD-NT but not full-length GSDMD and promotes pore formation (76, 77). GSDMD palmitoylation is primarily mediated by two palmitoyl acyl transferases (PAT), ZDHHC5 and ZDHHC9, and is regulated by ROS stress. Alanine mutation of Cys^191^/Cys^192^ and inhibition of GSDMD palmitoylation by 2-bromopalmitate (2-BP) or the GSDMD palmitoylation-specific competitive peptide (CPP-W) can effectively abrogate membrane localization, pyroptosis, and IL-1β release without affecting upstream transcription and cleavage of GSDMD. Furthermore, in a mouse sepsis model, inhibition of GSDMD palmitoylation alleviated organ injury and extended the survival of septic mice (76). Synoviolin (SYVN1), one of the RING E3 ligases, was shown to promote canonical and noncanonical inflammasome-induced pyroptosis by regulating GSDMD ubiquitination. SYVN1 interacts with GSDMD and ubiquitinates GSDMD at Lys^203^ and Lys^204^ residues with K27-linked polyubiquitin chains (78). Another type of posttranslational modification, GSDMD oxidation, has been shown to play an important role in regulating the cleavage of GSDMD and pore formation (79, 80). After NLRP3 inflammasome activation, mtROS oxidatively modifies Cys^38^, Cys^56^, Cys^268^ and Cys^467^ of human GSDMD (Cys^39^, Cys^57^, Cys^265^ and Cys^487^ of mouse GSDMD), thereby enhancing the release of GSDMD-NT domain and NLRP3 inflammasome-dependent pyroptosis (79). In addition, a recent report revealed that GSDMD oxidation at Cys^192^ by ROS promotes GSDMD oligomerization, pore formation, and pyroptosis (80).

In recent years, the virulence mechanisms of some pathogens that maintain infection by preventing GSDMD-induced pyroptosis have been successively revealed. The 3C-like protease nonstructural protein 5 (Nsp5) from coronaviruses (CoVs), including SARS-CoV-2, Middle East respiratory syndrome coronavirus (MERS-CoV), porcine deltacoronavirus (PDCoV), and porcine epidemic diarrhea virus (PEDV), can cleave porcine GSDMD at the Gln^193^-Gln^194^ junction into two inactive fragments, thereby inhibiting pyroptosis (81). The inhibition of pyroptosis by Nsp5 facilitates the replication of coronaviruses during the initial period to evade host immune responses. In addition to Nsp5, SARS-CoV-2 nucleocapsid protein also inhibits GSDMD-mediated pyroptosis. Mechanistically, SARS-CoV-2 nucleocapsid directly binds the GSDMD linker region to hinder the recognition and cleavage of the GSDMD tetrapeptide by caspase-1 (82). Furthermore, *Shigella* IpaH7.8, a bacterial ubiquitin ligase, was identified as an inhibitor of GSDMD-dependent pyroptosis and sustains pathogen infections by targeting human GSDMD-NT domain and ubiquitinating GSDMD for proteasomal degradation (83).

### 3.3 GSDMD pore formation and repair

A growing number of studies have revealed the regulatory mechanism of GSDMD membrane pore formation. In LPS-induced noncanonical inflammasome activation and pyroptosis pathways, guanylate-binding proteins (GBPs) have been shown to control multiple key steps (84). Among them, Gbp2 is responsible for recruiting caspase-11 for cytosolic LPS recognition and activation. After GSDMD cleavage by caspase-11, Gbp3 facilitates GSDMD-NT assembly and trafficking to form pores (84). In 2022, Liu and colleagues reported that tyrosine phosphatase B (PtpB) from *Mycobacterium tuberculosis* (Mtb) impairs GSDMD-NT membrane localization and pore formation to evade host GSDMD-dependent immunity and facilitate Mtb intracellular survival (85). Mechanistically, the Ub-interacting motif (UIM)–like region of PtpB binds to host ubiquitin through hydrophobic interactions. Then, the activated PtpB-Ub complex dephosphorylates phosphatidylinositol-4-monophosphate and phosphatidylinositol- (4, 5)-bisphosphate, leading to a significant decrease in the abundance of these phosphoinositides in host cell membranes, thereby disrupting the membrane localization of GSDMD-NT and inhibiting pyroptosis (85).

**Figure 3:**
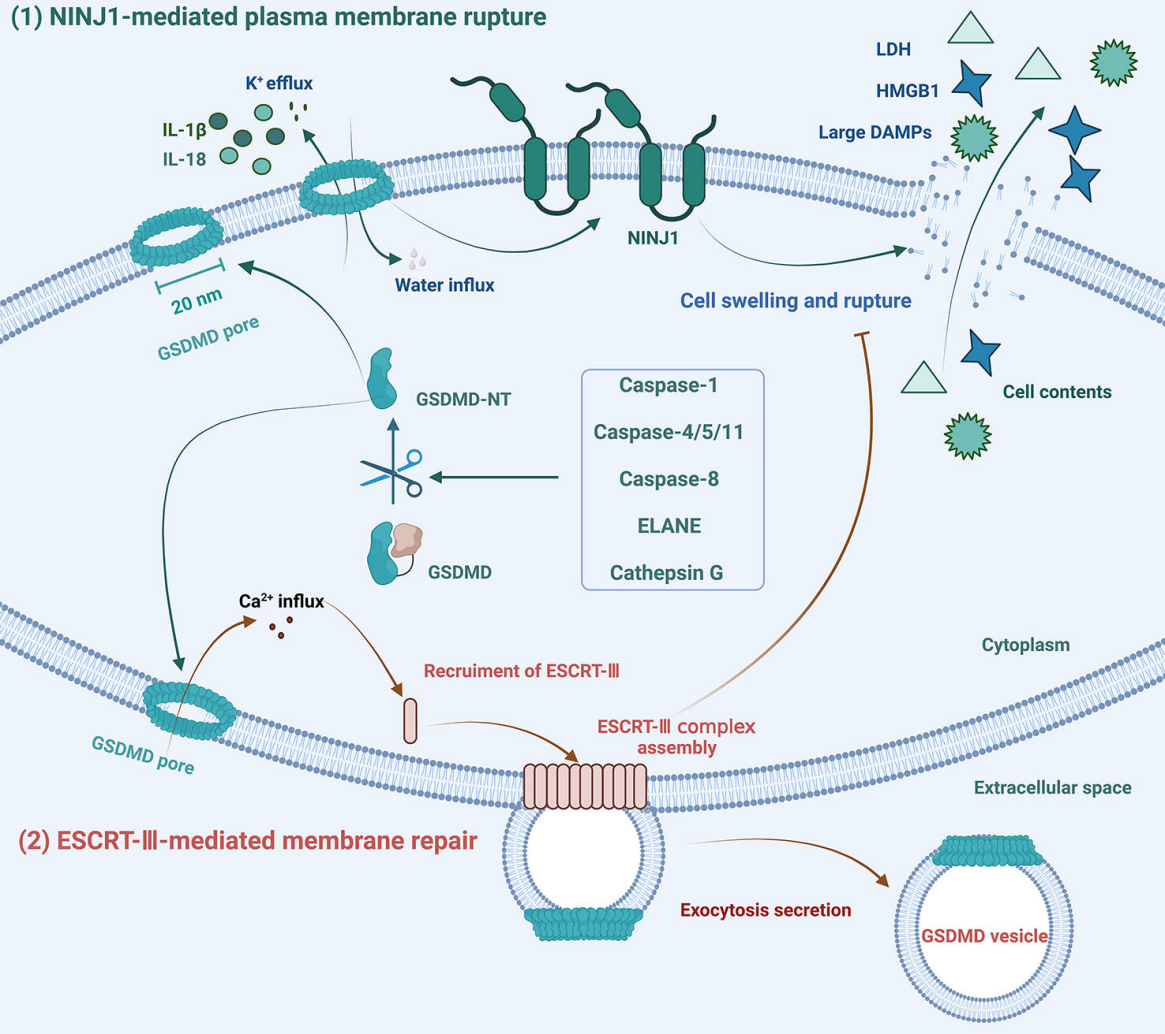
Schematic diagram of the mechanisms of formation and repair of GSDMD pores. (1) NINJ1 oligomerization licenses plasma membrane rupture and cell pyroptosis downstream of GSDMD pore formation. (2) In response to Ca^2+^ influx, ESCRT-III assembly is initiated and dynamically repairs GSDMD pores by exocytosis secretion to maintain membrane integrity.

Cell pyroptosis is a type of lytic cell death that eventually leads to plasma membrane rupture (PMR) and promotes inflammatory responses. The processes of PMR and repair of GSDMD pores are tightly regulated, as shown in **Figure 3**. Dixit and his colleagues discovered that ninjurin-1 (NINJ1), a 16-kDa cell surface protein with two transmembrane regions, plays an important regulatory role in PMR, revealing that cell death-driven PMR is an active event rather than a passive process (86). In unstimulated BMDMs, NINJ1 dimerizes or trimerizes, and it further oligomerizes in response to cell death signals. NINJ1 deficiency significantly reduces the release of GSDMD-related macromolecular proteins, including LDH and HMGB1, without affecting IL-1β secretion. This indicates that NINJ1 is indispensable for PMR, the final step of cell pyroptosis, but does not affect the formation and integrity of GSDMD pores (86).

Maintenance of plasma membrane integrity (PMI) is essential for cell survival and normal cellular function. Massive GSDMD membrane pores can cause severe loss of PMI and lytic cell death, but not all pore formation leads to pyroptosis. Cells with membrane pore repair mechanisms can tolerate limited plasma membrane damage. Several studies have shown that ESCRT-III can repair membrane damage caused by GSDMD pores, phosphorylated-mixed lineage kinase domain-like protein (pMLKL), detergents, pore-forming toxins, perforin, or laser light (87–89). Various forms of cell membrane damage, including damage caused by GSDMD pore formation, can result in Ca^2+^ influx, which then triggers the ESCRT repair program, leading to the shedding of damaged plasma membranes in the form of exosomes (88). Furthermore, the ESCRT system only performs the membrane repair process against cell lysis without affecting the activation of GSDMD (88).

In addition to restoring membrane integrity, the cell membrane repair mechanism also triggers PMI responses (90). Following sub-lethal plasma membrane damage, Ca^2+^ influx recruits Ser^660^-phosphorylated protein kinase C (PKC), which activates the downstream NF-κB signaling pathway and the RelA/Cux transcription factors to promote the expression and secretion of chemokines, such as CXCL1 and CXCL10. The massive release of chemokines recruits macrophages, converting cell membrane damage signals into immune signals to alert the surrounding microenvironment (90). Thus, the formation of GSDMD pores is in dynamic equilibrium with the membrane repair protein ESCRT-III. Cell pyroptosis or hyperactivation depends on the magnitude of the danger signals and the number of GSDMD pores. Under low-concentration stimulation, a limited level of cell membrane damage can be repaired, and the cell can survive danger signals.

### 3.4 Physiological functions of GSDMD

In addition to executing pyroptosis and inflammation, the physiologic function of GSDMD has been revealed by Wang’s group in 2022. Specifically, epithelial GSDMD maintains intestinal system homeostasis by regulating the secretion of mucin from goblet cells and the formation of mucus layers that spatially isolate the gut microbiota from colonic epithelial cells (91). The researchers detected significant GSDMD expression and activation in the gut, which is regulated by commensal microbiota and the NLRP6 inflammasome. In mice with the specific knockout of intestinal epithelial cells (GSDMD^ΔIEC^), the mucous layers in the intestine almost disappeared, accompanied by severe bacterial infection. The mechanism studies have shown that GSDMD pores-driven calcium ion influx activates scinderin-mediated F-actin breakdown and mucus secretion, leading to mucin granule exocytosis and mucus layer formation (91). It is worth mentioning that GSDMB, another member of the gasdermin family, has been proven to play a significant protective role in IBD independent of pyroptosis. In a recent study, GSDMB was found to repair epithelial barrier function and reduce inflammation by promoting the proliferation, migration, and adhesion of intestinal epithelial cells (IECs) (92). These findings show the important role of the gasdermin family in maintaining the homeostasis of the intestinal system, and other physiological functions of GSDMD remain to be explored.

## 4 GSDMD-driven diseases

GSDMD-induced pyroptosis plays an important role in defending against pathogen infection and DAMPs stimulation by facilitating the removal of infected or damaged cells. However, persistent GSDMD-mediated pyroptosis can lead to ion flow disturbances, organelle dysfunction, and excessive inflammatory responses, and is involved in the onset and development of a variety of diseases, including COVID-19 (93), HIV infection (30), neurodegenerative diseases (94), metabolic diseases (26), cancers (27, 29), IBD, and others (95).

### 4.1 Infectious diseases

COVID-19, caused by SARS-CoV-2 infection, has caused serious life-threatening and heavy economic burdens for humans. As of the time this manuscript was submitted, over 764 million confirmed cases and more than 6.9 million deaths had been reported globally, with case numbers continuing to rise (96). Severe SARS-CoV-2 infection can result in a range of symptoms, including dysregulated cytokines release, pneumonia, acute kidney injury (AKI), which can rapidly progress to acute respiratory distress syndrome (ARDS), disseminated intravascular coagulation, multisystem failure, and even death (93, 97). SARS-CoV-2 invades host cells by Fcγ receptors in monocytes or angiotensin-converting enzyme 2 (ACE2) in epithelial cells, which induces inflammasome activation and downstream GSDMD-mediated pyroptosis through multiple pathways (98, 99). Open reading frames 3a (ORF3a) of SARS-CoV-2 activates the NLRP3 inflammasome by inducing potassium efflux and mitochondrial ROS production (100). Both nucleocapsid (N) proteins and ORF8b can directly bind to NLRP3, leading to NLRP3 inflammasome activation and cytokine production (101, 102). In addition, non-structural protein 6 (NSP6) of SARS-CoV-2 interacts with ATP6AP1, a subunit of the lysosomal proton pump v-ATPase, to inhibit the lysosome autophagy system, thereby activating the NLRP3 inflammasome-mediated pyroptosis pathway (103, 104). In addition to NLRP3 inflammasome, AIM2 inflammasome and caspase-11/4 were also activated during SARS-CoV-2 infection (98, 105). The activation of both these canonical and noncanonical inflammasomes triggers GSDMD cleavage and pyroptosis, which in turn activates inflammasomes and amplifies inflammatory signaling (98, 99). Inflammasome activation and pyroptosis form a positive feedback loop, which leads to abnormal activation of the immune system, resulting in a severe inflammatory cascade and cytokine storm, also known as cytokine release syndrome (CRS) (106). In severe cases of COVID-19, the levels of serum LDH and inflammatory factors, particularly IL-6, TNF-α, IL-1β, and IL-18, were significantly increased, suggesting the occurrence of pyroptosis and other forms of necrotic cell death (98, 99, 107). The main causes of cytokine storm are considered to be extensive pyroptosis and subsequent recruitment of immune cells, which lead to excessive tissue inflammation, organ failure, and even death. Therefore, blocking GSDMD-mediated pyroptosis and cytokine storm is a promising strategy to ameliorate severe COVID-19.

Excessive pyroptosis is also involved in sepsis, a common complication of infection. Deletion of GSDMD has been shown to protect against sepsis and improve survival in mouse models of sepsis, indicating the critical role of GSDMD in sepsis (39, 108–110). LPS-induced sepsis is predominantly associated with noncanonical inflammasome activation (39), and deficiency of caspase-11 or GSDMD protects from sepsis-induced death (39, 49, 69, 109). A recent study revealed that sepsis-derived S100A8/A9 induces GSDMD-dependent platelet pyroptosis in severe sepsis by upregulating the TLR4/NLRP3 signaling pathway, which leads to the release of oxidized mtDNA and promotes NETs formation (111). Moreover, NETs were shown to release S100A8/A9 to further induce GSDMD-dependent platelet pyroptosis, forming a deleterious positive feedback loop that exacerbates the inflammatory response after infection (111). Several GSDMD inhibitors, including disulfiram, necrosulfonamide (NSA), and dimethyl fumarate (DMF), have been shown to be effective in relieving sepsis (75, 108, 109). This suggests that the blockade of the GSDMD-related pyroptotic pathway could be a potential therapeutic for sepsis.

### 4.2 Neurodegenerative diseases

The pathogenesis of neurodegenerative diseases is a complex process in which neuroinflammation is considered to be a significant driving force. The link between GSDMD-related inflammation and neurodegenerative diseases has been highlighted since the mechanism of pyroptosis was revealed. Alzheimer’s disease (AD) is the most prevalent neurodegenerative disease worldwide, with the dual pathological features of neuroinflammatory plaques formed by Aβ aggregation and neurofibrillary tangles (NFTs) composed of hyperphosphorylated tau (p-tau). Clinical manifestations of AD patients include obvious memory loss, cognitive decline, language difficulties, movement disorders, emotional changes, etc. Pyroptosis is involved in early Aβ deposition and neuronal death, promoting the occurrence and progression of AD (112, 113). The caspase-1 inhibitor VX-765 was shown to reduce neuronal death with significant protective effects in mouse models of AD (114), and NLRP3 deficiency also significantly reduces Aβ aggregation and cognitive impairment (115–117). Therefore, inhibiting the inflammasome and pyroptosis pathways is a potential strategy for alleviating and treating AD.

GSDMD-mediated pyroptosis has also been implicated in the pathogenesis of Parkinson’s disease (PD), the second most common neurodegenerative disease characterized by the presence of lewy bodies rich in α-synuclein aggregates, loss of dopaminergic neurons, and motor dysfunction. In a mouse model of PD induced by *N*-methyl-4-phenyl-1,2,3,6-tetrahydropyridine (MPTP), Shi et al. demonstrated that baicalein inhibited nigral dopaminergic neuron death, glial activation, and motor dysfunction by blocking the NLRP3-caspase-1-GSDMD pathway and reducing neuroinflammation (33). A recent study showed that Prussian blue nanozyme (PBzyme) inhibited the upstream ROS-NLRP3-GSDMD pathway and microglia pyroptosis, thereby reducing dopaminergic degeneration, neuroinflammation, and motor dysfunction in the MPTP-induced PD mouse model (118).

### 4.3 Cancer

Pyroptosis, induced by the gasdermin family, is a form of lytic cell death that can inhibit the development of cancer by inducing tumor cell death. Several studies have revealed the role of different members of the gasdermin family, such as GSDMB, GSDME, and GSDMD, in antitumor immunity (9, 119, 120). While GSDMD-dependent pyroptosis has been shown to promote tumor cell death and inhibit the proliferation of gastric cancer cells (121), its role in other types of tumors is more complex. Cucurbitacin B, a natural bioactive product extracted from the muskmelon pedicel, was reported to exert anti-lung cancer activity by activating TLR4/NLRP3/GSDMD-driven pyroptosis in non-small cell lung cancer (NSCLC) cells and mouse models (122). Paradoxically, another study showed that inhibition of GSDMD expression inhibited tumor cell proliferation in NSCLC (29). In addition, overexpression of GSDMD also promotes tumor proliferation in bladder cancer (123). Therefore, GSDMD-mediated pyroptosis is a double-edged sword for tumor development, and the detailed mechanisms of whether it promotes or inhibits tumor survival in different tissues and genetic backgrounds need further exploration.

### 4.4 Metabolic diseases

Diabetic nephropathy (DN) is a common complication of type 2 diabetes (T2D), which eventually leads to renal failure and end-stage renal disease (ESRD). In a high-fat diet (HFD)/streptozotocin (STZ)-induced diabetic mouse model, GSDMD-mediated pyroptosis was activated and involved in the development of DN. Knockdown of caspase-11/4 or GSDMD significantly alleviated symptoms in diabetic mice (124, 125). Some active substances, such as hirudin and punicalagin, have been shown to attenuate diabetic nephropathy and ameliorate kidney damage in mice by inhibiting the GSDMD-mediated pyroptosis pathway (125, 126).

Nonalcoholic steatohepatitis (NASH) is a progressive form of nonalcoholic fatty liver disease (NAFLD) characterized by toxic lipid accumulation in the liver and liver inflammation. The role of GSDMD in the pathogenesis of steatohepatitis has been revealed. GSDMD and its active fragment GSDMD-NT have been found to be up-regulated in the liver tissues of NAFLD/NASH patients (26). In addition, significantly elevated GSDMD-NT protein levels in the liver positively correlated with the NAFLD activity score (NAS) and progression of fibrosis. In methionine-and-choline-deficient (MCD)-fed mice, knockout of GSDMD reduced liver triglycerides and significantly alleviated liver inflammation and liver fibrosis, suggesting that GSDMD plays an important role in the development of steatohepatitis (26). Furthermore, GSDMD-mediated pyroptosis is also activated in alcoholic hepatitis (AH). The deficiency of caspase-11 or GSDMD prevents membrane pore formation and excessive IL-1β secretion, thereby ameliorating alcohol-induced liver injury (127, 128).

### 4.5 Other inflammatory diseases

Inflammatory bowel disease (IBD), including ulcerative colitis (UC) and Crohn’s disease (CD), is associated with the disruption of intestinal epithelial barrier. Dextran sulfate sodium (DSS) is a commonly used inducer for the construction of acute ulcerative colitis models. In a DSS-induced IBD model, the NLRP3 inflammasome has been shown to be involved in the development of experimental IBD and plays an important role in protecting the integrity of the intestinal mucosal barrier (129). The expression of GSDMD is increased in IECs from both DSS-induced colitis mice and IBD patients, and GSDMD deficiency effectively reduces the severity of DSS-induced colitis (95, 130). Paradoxically, Ma et al. discovered an unexpected physiological role for GSDMD in experimental colitis. GSDMD in macrophages but not epithelial cells protects against DSS-induced colitis, and GSDMD deficiency causes more severe DSS colitis (131). These seemingly controversial findings suggest that GSDMD may have different functional mechanisms in different cell types and under different experimental conditions.

Familial Mediterranean fever (FMF), the most common monogenic autoinflammatory disease worldwide, is caused by mutations in the MEFV gene that encodes the pyrin protein. MEFV mutations predispose the pyrin inflammasome to activation, leading to uncontrolled pyroptosis, fever, and pain. In a mouse model of FMF, GSDMD deficiency protects against autoinflammatory diseases (21). Dimethyl fumarate (DMF) inhibits pyroptosis by covalently modifying GSDMD and effectively alleviates weight loss and splenomegaly symptoms in a MEFV mutation-induced FMF mouse model (75).

Experimental autoimmune encephalitis (EAE), a type of autoinflammatory disease in the central nervous system, is the most commonly used animal model to study multiple sclerosis (MS), with the main pathological features of MS such as axonal demyelination and neuroinflammation. GSDMD is essential for EAE induction, and its deficiency inhibits the infiltration of immune cells into the CNS, improving neuroinflammation and demyelination in a mouse model of EAE (23). VX-765, a caspase-1 inhibitor, reduces neuroinflammation and axonal damage in an EAE model (132). Disulfiram and DMF, two GSDMD inhibitors, have been shown to inhibit the onset of EAE and significantly reduce neuropathology and clinical and histopathological scores (23, 75).

Neonatal-onset multisystem inflammatory disease (NOMID) is the most severe form of familial cold autoinflammatory syndrome (FCAS), caused by mutations in the CIAS1 gene encoding NLRP3 protein (133). Studies have shown that GSDMD-driven pyroptosis is required for NOMID pathogenesis in mice. In NOMID cells and mice, deletion of GSDMD can effectively block NOMID-associated inflammatory symptoms, relieve symptoms such as systemic inflammation and organ damage, and prolong survival (22).

## 5 GSDMD inhibitors

**Figure 4:**
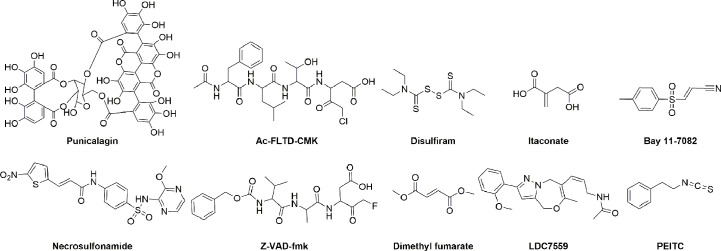
The structure of GSDMD inhibitors.

As mentioned above, inhibition or deletion of GSDMD has been shown to have a protective effect on a variety of animal models of inflammatory diseases, such as sepsis and viral infections. Therefore, inhibition of the GSDMD-mediated pyroptosis pathways is an effective strategy to alleviate and treat inflammatory disease. Some GSDMD inhibitors have been reported, and their chemical structures are shown in **Figure 4**. These inhibitors were divided into indirect GSDMD inhibitors and direct GSDMD inhibitors based on their molecular mechanisms.

### 5.1 Indirect GSDMD inhibitors

Given the structural characteristics of the GSDMD cleavage site (peptide FLTD) and the caspase inhibitor containing a halomethyl ketone group, *N*-acetyl-Phe-Leu-Thr-Asp-chloromethylketone (Ac-FLTD-CMK) was developed to specifically inhibit inflammatory caspases without affecting apoptotic caspase-3 (134). Ac-FLTD-CMK inhibited the *in vitro* activities of inflammatory caspase-1, -4, and -5 with IC_50_ values of 46.7 nM, 1.49 μM, and 0.329 μM, respectively, thereby inhibiting the cleavage of GSDMD, pyroptosis, and the release of cytokines (134). The crystal structure of the Ac-FLTD-CMK and caspase-1 complex (PDB ID: 6BZ9) showed that Ac-FLTD-CMK binds to the catalytic groove of the caspase-1 p10/p20 heterodimer through hydrophobic interactions and hydrogen bonding. In addition, the covalent bond formed by the CMK fragment and Cys^285^ of caspase-1 enhanced the docking of Ac-FLTD-CMK to caspase-1. The formation of the Ac-FLTD-CMK and caspase-1 complex blocks the recognition of GSDMD by caspase-1, thereby inhibiting GSDMD activation and pyroptosis. Another study showed that Ac-FLTD-CMK reduced GSDMD protein expression and production of cytokines in a murine model of lupus nephritis, thus reducing glomerulosclerosis and immune cell infiltration and preventing the development of lupus nephritis (135). Z-VAD-fmk, another non-specific caspase inhibitor, blocks inflammatory caspases 1/4/5/11-mediated GSDMD activation and the activity of caspases 3/7/8, thus inhibiting cell pyroptosis and apoptosis (134, 136, 137).

Punicalagin, a polyphenolic compound extracted from pomegranate, has been shown to block phosphatidylserine flip and stabilize lipids, thereby inhibiting membrane permeability and IL-1β release with IC_50_ values of 7.65 and 3.91 μM, respectively. The interference of punicalagin on membrane fluidity may prevent the insertion of GSDMD-NT into the plasma membrane, and its pharmacological effect is similar to that of GSDMD inhibitors, but the direct effect of punicalagin on GSDMD still needs further studies to clarify (138).

Although extracellular addition of glycine has been shown to inhibit gasdermin-mediated cell lysis by regulating osmotic pressure, it does not prevent intracellular processes leading to cell death or IL-1β release. Thus, glycine is not a specific inhibitor of GSDMD-related pore formation and pyroptosis (139). In addition, lanthanide ions, including La^3+^ and Gd^3+^, Mg^2+^ ion, and hypertonic solutions can also inhibit membrane rupture through a non-specific mechanism (140, 141).

### 5.2 Direct GSDMD inhibitors

Specific inhibition of the GSDMD protein is considered a more precise and desirable therapeutic strategy. Mutation studies have revealed several potential targets for GSDMD, of which Cys^191^ (mouse Cys^192^) has an important role in pore formation and is by far the most studied active site. Mutations in Leu^192^ inhibit the binding of GSDMD-NT to membrane lipids, and covalent modification of the Cys^191^ site is likely to have a similar effect on GSDMD (142). As listed in **Supplementary Table 1**, the development of specific GSDMD inhibitors is still in its infancy, and most inhibitors block pyroptosis by covalently modifying cysteine Cys^191^ (mouse Cys^192^) of GSDMD.

NSA was initially discovered during screening for human mixed lineage kinase domain-like pseudokinase (MLKL) inhibitors to block MLKL-associated necroptosis by binding to Cys^86^ of MLKL to disrupt disulfide bonds (143). In 2018, Abbott et al. identified NSA as the first inhibitor to directly target GSDMD (108). NSA can bind to GSDMD with a K_D_ value of 32 μM and inhibit the pyroptosis induced by the activation of inflammasomes such as NLRP3, NLRC4, and pyrin (**Supplementary Table 1**). NSA does not affect the activation of GSDME, another member of the gasdermin family. Treatment with NSA significantly reduces inflammatory cytokine release and prolongs survival in mice with sepsis. However, other studies have shown that NSA can also inhibit the upstream pathways of GSDMD-mediated pyroptosis, such as LPS-induced gene transcription and activation of caspase-1 (109, 144). Therefore, NSA is a direct but nonspecific GSDMD inhibitor.

Disulfiram (DSF), also known as Antabuse, is an FDA-approved drug used to treat alcohol addiction by inhibiting aldehyde dehydrogenase (145). Recently, Wu’s group discovered the efficacy of DSF in inhibiting pyroptosis by high-throughput screening for GSDMD-related pyroptosis inhibitors using a liposome leakage assay (109). DSF bound to GSDMD with a K_D_ of approximately 12.8 μM and blocked pyroptosis downstream of canonical and non-canonical inflammasome activation, with no effect on necroptosis. The addition of copper gluconate can significantly increase the inhibitory activity of DSF and its metabolite diethyldithiocarbomate (DTC) on pyroptosis. Disulfiram chelating with Cu(II) is 24-fold more potent in inhibiting NLRP3 inflammasome-mediated pyroptosis, with an IC_50_ of 0.41 ± 0.02 μM. Mass spectrometry analysis and mutation studies revealed that DSF covalently modified Cys^191^ (mouse Cys^192^) of GSDMD to inhibit GSDMD pore formation. In addition, DSF had an inhibitory effect on upstream NF-κB and inflammatory caspases (146). The IC_50_ values of DSF for inhibiting recombinant caspase-1 and caspase-11 *in vitro* were 0.15 ± 0.04 μM and 0.73 ± 0.07 μM, respectively. There is some evidence to suggest that disulfiram’s effect of inhibiting pyroptosis is mainly attributable to its inhibition of GSDMD pore formation rather than suppressing GSDMD cleavage or other upstream events (109). Similar to NSA, DSF improved mortality in an LPS-induced sepsis model at lethal doses. The therapeutic role of DSF in EAE has also been demonstrated, and disulfiram treatment is effective in preventing the development of EAE (23). Additionally, disulfiram has been found to reduce the incidence and severity of COVID-19 and is currently being evaluated in two phase II clinical trials (NCT04485130 and NCT04594343) for the treatment of COVID-19 (147).

DMF, an FDA-approved drug (Tecfidera) for the treatment of multiple sclerosis, has been proven to inhibit GSDMD by Fitzgerald et al. (75). DMF blocks pyroptosis induced by NLRP3, NLRC4, or AIM2 inflammasome activation, and endogenous fumarate accumulation also reduces GSDMD-NT formation and cell lysis. Mechanistic studies have shown that DMF succinates Cys^191^ of human GSDMD (mGSDMD Cys^192^) and other cysteines (Cys^56,^ Cys^268^, Cys^309^, and Cys^467^ in human; or Cys^39^, Cys^57^, Cys^77^, Cys^122^, Cys^265^, Cys^299^, Cys^434^, Cys^448^, and Cys^489^ in mouse) and blocks the caspase-1-GSDMD interaction, thereby inhibiting GSDMD cleavage, GSDMD-NT oligomerization, pyroptotic pore formation, and cell lysis. Another study, however, showed that DMF had no effect on GSDMD-induced liposome leakage (109). In addition to binding GSDMD, DMF covalently modified the key amino acid Cys^45^ and other cysteines of GSDME, inhibiting GSDME-dependent pyroptosis. The protective effects of DMF in various disease models have also been validated. In septic shock induced by lethal doses of LPS, DMF treatment significantly reduced the release of the inflammatory cytokine IL-1β and improved survival in mice. GSDMD-NT and inflammatory cytokines were elevated in the EAE model, and GSDMD deficiency protected mice from EAE as previously described (23). DMF can effectively reduce GSDMD-NT and IL-1β in central nervous system tissues, immune cells infiltration, and hindered the progression of EAE disease. Besides this, in the Mefv^V726^/^V726^ mouse model, DMF alleviated symptoms of mice with FMF disease such as IL-1β release, weight loss, and splenomegaly (75).

BAY 11-7082, described as an NF-κB inhibitor, was recently discovered to inhibit both classical and non-classical inflammasome-driven pyroptosis (146). Mass spectrometry analysis showed that BAY 11-7082 covalently modified Cys^191^ of GSDMD, effectively inhibiting GSDMD pore formation, IL-1β secretion, and pyroptosis. BAY 11-7082 bound directly to GSDMD with a K_D_ of 35.6 μM and moderately inhibited caspase-11-mediated liposome leakage with an IC_50_ of 6.81 μM (**Supplementary Table 1**). In addition, BAY 11-7082 significantly inhibited inflammatory caspase-1 and caspase-11 with IC_50_ values of 0.15 μM and 1.96 μM, respectively. The inhibition of liposome leakage by BAY 11-7082 is thought to be mainly due to the inhibition of caspase-1/11. Overall, BAY 11-7082 is a non-specific GSDMD inhibitor that inhibits multiple steps of the pyroptotic pathway, including NF-κB activation and caspase-1 processing, thereby inhibiting GSDMD cleavage, IL-1β release, and cellular rupture (146).

LDC7559 with the pyrazolo-oxazepine scaffold was previously reported to inhibit GSDMD-mediated pyroptosis by binding to GSDMD, thereby inhibiting the formation of NETs, with IC_50_ values of ~5.6 μM in PMA-induced NETosis and ~300 nM in cholesterol crystal-induced NETosis in murine neutrophils (**Supplementary Table 1**) (26). However, a more recent report indicated that LDC7559 indeed inhibited the formation of NETs and NETosis as previously described, but that this inhibition was independent of GSDMD. The exact molecular biology mechanism of LDC7559 is unclear (109).

Itaconate, an intracellular metabolite with a Michael receptor structure, has previously been shown to inhibit the activation phase of NLRP3 inflammasome (148). Artyomov et al. revealed the direct post-translational modification of GSDMD by endogenous itaconate (149). After prolonged inflammatory stimulation, aconitate decarboxylase 1 induces massive accumulation of endogenous itaconate, which prevents full activation of caspase-1 and cleavage of GSDMD, thereby enhancing cellular tolerance to prolonged LPS exposure. Mass spectrometry analysis has revealed that endogenous itaconate directly bound to GSDMD through covalent modification of α, β-unsaturated double bonds with sulfhydryl groups of GSDMD Cys^77^ and potentially interfered with the caspases-GSDMD interaction, thereby inhibiting pyroptosis (149).

Phenethyl isothiocyanate (PEITC), a secondary metabolite of cruciferous vegetables, was identified as a covalent inhibitor of GSDMD by Xu et al. (150). PEITC reduces NLRP3 production and cleavage of caspase-1 and GSDMD in a mouse model of acute liver injury and in AML12 cells *in vitro*. Mechanistic studies have shown that PEITC binds to human WT-GSDMD but not to the C191A-GSDMD mutant with a Kd of 230 nM. Due to the high reactive affinity of the –N=C=S group for cysteine, PEITC inhibits pyroptosis by covalently modifying Cys^191^ of GSDMD. In addition, PEITC can substantially attenuate concanavalin A (ConA)-induced inflammatory liver injury and carbon tetrachloride (CCl_4_)-induced chemical liver injury in a dose-dependent manner by inhibiting hepatocyte pyroptosis (150).

The discovery and pharmacological studies of the above inhibitors show that the GSDMD-related pyroptosis pathway can be effectively inhibited, confirming that the GSDMD protein is a potential drug target. Only a few direct GSDMD inhibitors, including NSA, DSF, and DMF, etc., have been currently discovered. These inhibitors inhibit pyroptosis and downstream inflammation mainly by covalently modifying the residue Cys^191^. However, many proteins *in vivo* have reactive sulfhydryl groups. In addition to upstream caspases and GSDMD, the covalent modification of sulfhydryl groups in multiple targets *in vivo* may lead to toxic side effects. Peptide inhibitors are another class of inhibitors, but their poor druggability due to poor membrane permeability and easy degradation *in vivo* presents a challenge. Despite these limitations, the development of effective and specific GSDMD inhibitors is still worth pursuing, given the important role of GSDMD-mediated pyroptosis in various inflammatory diseases.

## 6 Perspective conclusions

An increasing number of pyroptosis-related studies have advanced our understanding of the structure, function, and activation mechanisms of GSDMD. The activation process of GSDMD is tightly regulated by multiple pathways. PAMPs and DAMPs induce activation of canonical or noncanonical inflammasomes, thereby triggering GSDMD activation and pyroptosis. In addition to inflammatory caspases, apoptotic caspase-8, cathepsin G, and ELANE are also involved in GSDMD cleavage and pore formation. The negative regulatory mechanisms of GSDMD that ensure its correct and beneficial activation have also been reported in recent years. Both apoptotic caspase-3 and caspase-7 cleave GSDMD at Asp^87^, independent of active sites, resulting in the formation of an inactive N-terminal fragment (p45) that specifically blocks GSDMD activation. This crosstalk between apoptosis and pyroptosis suggests a complex interplay between cell death pathways in the innate immune system. In addition, the formation of GSDMD pores does not always lead to necrosis or cell lysis. Negative regulatory mechanisms have also been found in the downstream events of GSDMD-dependent pyroptosis. GSDMD plasma membrane pores can be dynamically repaired by ESCRT-III to delay or block the process of pyroptosis. The degree of inflammation in the cells and the size and number of pores may determine whether GSDMD pores are repaired or lysed. Collectively, numerous studies have reported mechanisms of inflammatory activation and inflammatory diseases, but little is known about negative regulatory mechanisms such as inflammation inactivation. In addition to its protective roles in innate immune defense and septic shock, the physiological function of GSDMD in epithelial cells to maintain intestinal mucosal homeostasis has recently been discovered. This suggests that the fate of GSDMD varies in different cell types and in different physiological or pathological environments. And whether GSDMD has any other physiological functions remains to be explored.

GSDMD has been shown to play an important role in various inflammatory diseases. Inhibition of GSDMD activation, membrane pore formation, and downstream inflammatory responses can precisely control the degree of inflammation without disrupting upstream inflammasome activation. Induction of apoptosis rather than pyroptosis can suppress pathogenic microbial infection and trigger a modest inflammatory response, thereby avoiding extensive tissue inflammation. Therefore, the development of highly specific GSDMD inhibitors is conducive to maintaining a moderate inflammatory response and provides a promising therapeutic strategy for nonresolving inflammatory diseases. A few inhibitors that target GSDMD mainly inhibit pyroptosis by covalently modifying the cysteine Cys^191^ (murine Cys^192^) of the GSDMD protein. However, these covalent inhibitors lack specificity, and unknown risks of toxicity limit their further clinical application. Therefore, the discovery of GSDMD inhibitors and their application in the treatment of related inflammatory diseases remains a challenge. The crystal structures of caspase-1-GSDMD and caspase-GSDMD-CT complexes reveal the structural basis for the highly specific recognition of the substrate GSDMD by caspases, guiding the design and development of GSDMD inhibitors that target the critical binding site. Furthermore, molecular mechanism studies of pore formation and cell lysis downstream of pyroptosis may provide additional therapeutic strategies for pharmacological inhibition of pyroptosis-related diseases, such as targeting NINJ1.

Overall, many questions related to GSDMD-dependent pyroptosis remain unanswered. For example, do cleaved GSDMD-CT fragments have physiological or pathological activity? How do other proteases such as apoptotic caspases, ELANE, and cathepsin G recognize and cleave GSDMD substrates? Besides, the role and mechanism of GSDMD in disease models need to be further explored and validated. The development of non-covalent inhibitors targeting GSDMD is of great significance for regulating inflammatory responses. Therefore, more efficient GSDMD inhibitors are urgently needed to be discovered and developed.

## Author contributions

ZD wrote the manuscript and made figures. W-CL, X-YC, XW, and J-LL discussed and revised the manuscript. XZ designed and revised the manuscript. All authors contributed to the article and approved the submitted version.
