# Supplementary material for: Gasdermin D-mediated pyroptosis: mechanisms, diseases, and inhibitors
Source: Front Immunol. 2023 May 18;14:1178662. doi: 10.3389/fimmu.2023.1178662 (PMC10232970; doi:10.3389/fimmu.2023.1178662)
Supplement: Supplementary file 1 [file Table_1.docx]

Supplementary Material

Gasdermin D-mediated pyroptosis: mechanisms, diseases, and inhibitors

Zhen Dai^1*^, Wan-Cong Liu^1^, Xiao-Yi Chen^2^, Xiao Wang^2^, Jun-Long Li^1^, Xiang Zhang^1*^

**AUTHOR AFFILIATIONS:**

^1^Sichuan Industrial Institute of Antibiotics, School of Pharmacy, Chengdu University, Chengdu, China.

^2^ Key Laboratory of Drug Quality Control and Pharmacovigilance, Ministry of Education, China Pharmaceutical University, Nanjing, China.

*** Correspondence:** Zhen Dai: [daizhen15@163.com](mailto:daizhen15@163.com)

Xiang Zhang: XZhang_CDU@hotmail.com

**Supplementary Table 1.** Direct GSDMD inhibitors.

| Inhibitors | IC_50_ | Mechanisms | | GSDMD-Related Diseases | Clinical Studies^1^ | Ref. |
| --- | --- | --- | --- | --- | --- | --- |
| NSA | ~9.5 µM in GSDMD-dependent liposome leakage;  ~10-15 µM  in canonical inflammasome-induced pyroptosis | Covalently binds to Cys^191^ of GSDMD with a K_D_ of 32 μM, and blocks GSDMD-NT oligomerization and GSDMD pore formation | | Sepsis | Preclinical | (1, 2) |
| DSF | ~0.3 µM in GSDMD-mediated liposome leakage assay; ~7.7 µM in NLRP3 inflammasome-driven pyroptosis; ~10.3 µM in noncanonical inflammasome-driven pyroptosis | | Covalently binds to Cys^191^ of GSDMD with a K_D_ of 12.8 μM, and blocks GSDMD-NT oligomerization and GSDMD pore formation | Sepsis, EAE, Renal fibrosis;  COVID-19 | An FDA- approved drug for alcoholism (Antabuse)  phase II clinical trials (NCT04485130 and NCT04594343) for COVID-19 | (1, 3-5) |
| DMF | / | | Covalently modifies Cys^191^ and other cysteines of GSDMD and disrupts caspase-1-GSDMD interaction, thereby blocking GSDMD-NT oligomerization and GSDMD pore formation | Sepsis，  EAE, FMF, MS | An FDA- approved drug for MS(Tecfidera)  Phase II/Phase Ⅲ Clinical (NCT04381936) for COVID-19 | (1, 6) |
| BAY 11-7082 | ~6.8 µM in GSDMD-dependent liposome leakage | | Binds to GSDMD with a K_D_ of 35.6 μM, covalently modifies GSDMD Cys^191^, and inhibits inflammatory caspases and GSDMD cleavage | / | Preclinical | (1, 4) |
| LDC7559 | ~5.6 µM in PMA-induced NETosis; ~0.3 µM in cholesterol crystal-induced NETosis | | Binds to GSDMD, unclear molecular biology mechanism | / | Preclinical | (1, 7) |
| Itaconate | / | | Covalently modifies Cys^77^ of GSDMD and inhibits GSDMD cleavage | / | Preclinical | (8) |
| Ac-FLTD-CMK | 46.7 nM, 1.49 μM and 0.329 μM in inflammatory caspase-1, caspase-4, and caspase-5 activities | | Binds to inflammatory caspases and disrupts caspases-GSDMD interaction, thus inhibiting GSDMD-induced pyroptosis | Lupus nephritis | Preclinical | (9, 10) |
| PEITC | / | | Binds to GSDMD with a K_D_ of 230 nM, covalently modifies Cys^191^ of GSDMD, reduces the NLRP3 expression and inhibits the cleavage of caspase-1 and GSDMD | Acute liver injury | Phase Ⅰ clinical trial (NCT00968461 for lymphoproliferative disorder)  Phase II clinical trial (NCT00691132 for lung cancer) | (11) |

^1^Clinical studies registered at <https://www.cortellis.com/drugdiscovery>.

**References**

1. Hu JJ, Liu X, Xia S, Zhang Z, Zhang Y, Zhao J, et al. FDA-approved disulfiram inhibits pyroptosis by blocking gasdermin D pore formation. *Nat Immunol* (2020) 21(7):736-45. doi: 10.1038/s41590-020-0669-6.

2. Rathkey JK, Zhao J, Liu Z, Chen Y, Yang J, Kondolf HC, et al. Chemical disruption of the pyroptotic pore-forming protein gasdermin D inhibits inflammatory cell death and sepsis. *Sci Immunol* (2018) 3(26):eaat2738. doi: 10.1126/sciimmunol.aat2738.

3. Li S, Wu Y, Yang D, Wu C, Ma C, Liu X, et al. Gasdermin D in peripheral myeloid cells drives neuroinflammation in experimental autoimmune encephalomyelitis. *J Exp Med* (2019) 216(11):2562-81. doi: 10.1084/jem.20190377.

4. Hu JJ, Liu X, Zhao J, Xia S, Ruan J, Luo X, et al. Identification of pyroptosis inhibitors that target a reactive cysteine in gasdermin D. *BioRxiv* (2018):365908. doi: 10.1101/365908.

5. Zhang Y, Zhang R, Han X. Disulfiram inhibits inflammation and fibrosis in a rat unilateral ureteral obstruction model by inhibiting gasdermin D cleavage and pyroptosis. *Inflamm Res* (2021) 70(5):543-52. doi: 10.1007/s00011-021-01457-y.

6. Humphries F, Shmuel-Galia L, Ketelut-Carneiro N, Li S, Wang B, Nemmara VV, et al. Succination inactivates gasdermin D and blocks pyroptosis. *Science* (2020) 369(6511):1633–37. doi: 10.1126/science.abb9818.

7. Sollberger G, Choidas A, Burn GL, Habenberger P, Di Lucrezia R, Kordes S, et al. Gasdermin D plays a vital role in the generation of neutrophil extracellular traps. *Sci Immunol* (2018) 3(26):eaar6689. doi: 10.1126/sciimmunol.aar6689.

8. Bambouskova M, Potuckova L, Paulenda T, Kerndl M, Mogilenko DA, Lizotte K, et al. Itaconate confers tolerance to late NLRP3 inflammasome activation. *Cell Rep* (2021) 34(10):108756. doi: 10.1016/j.celrep.2021.108756.

9. Cao H, Liang J, Liu J, He Y, Ke Y, Sun Y, et al. Novel Effects of combination therapy through inhibition of caspase-1/gasdermin D induced-pyroptosis in lupus nephritis. *Front Immunol* (2021) 12:720877. doi: 10.3389/fimmu.2021.720877.

10. Yang J, Liu Z, Wang C, Yang R, Rathkey JK, Pinkard OW, et al. Mechanism of gasdermin D recognition by inflammatory caspases and their inhibition by a gasdermin D-derived peptide inhibitor. *Proc Natl Acad Sci U S A* (2018) 115(26):6792-97. doi: 10.1073/pnas.1800562115.

11. Wang J, Shi K, An N, Li S, Bai M, Wu X, et al. Direct inhibition of GSDMD by PEITC reduces hepatocyte pyroptosis and alleviates acute liver injury in mice. *Front Immunol* (2022) 13:825428. doi: 10.3389/fimmu.2022.825428.
